# Influence of Infraspinatus and Subscapularis Pathologies on Supraspinatus Muscle Atrophy - A Retrospective Cohort Study

**DOI:** 10.5704/MOJ.2007.007

**Published:** 2020-07

**Authors:** E Altan, A Nayman, A Yildirim, MU Ozbaydar, S Ciftci, M Karahan

**Affiliations:** 1 Department of Orthopaedics, Kadikoy Florence Nightingale Hospital - Beykent University Orthotics and Prosthetics Department, Istanbul, Turkey; 2 Department of Radiology, Selcuk University Medical Faculty Hospital, Konya, Turkey; 3 Department of Orthopaedics and Traumatology, Selcuk University Medical Faculty Hospital, Konya, Turkey; 4 Department of Orthopaedics and Traumatology, Acibadem University, Istanbul, Turkey; 5 Department of Orthopaedics and Traumatology, Acibadem Health Group, Istanbul, Turkey

**Keywords:** rotator cuff, tendons, shoulder, arthroscopy, magnetic resonance imaging

## Abstract

**Introduction:**

Many factors could affect the supraspinatus (SSP) muscle after tendon rupture. We aimed to determine how infraspinatus and subscapularis tendon problems affect supraspinatus muscle atrophy associated with tears, in a retrospective cohort study conducted in a tertiary-level centre.

**Material and Methods:**

Fifty-eight patients with a full-thickness SSP tendon tear who fulfilled the inclusion criteria were enrolled in the study. They were evaluated for tear retraction, fatty degeneration, and other rotator cuff tendon pathologies. Supraspinatus muscle was assessed using the Goutallier classification, and its average area was also measured. Accompanying lesions of the subscapularis and infraspinatus tendons and degree of supraspinatus muscle atrophy were evaluated using magnetic resonance imaging.

**Results:**

Our results showed that supraspinatus tendon tears ranged between 3mm and 41mm, and the estimated average cross-sectional area of the SSP muscle was 247.6mm^2^. Any degree of infraspinatus tendon pathology, ranging from tendinosis to full-thickness tears, was significantly correlated with the SSP muscle area (P < 0.05). The subscapularis tendon pathologies did not show a similar correlation. The interobserver and intraobserver reliabilities of the measurements were graded as excellent.

**Conclusion:**

Impairment of any of the rotator cuff muscles may affect the other muscles inversely. Our study showed that all infraspinatus tendon pathologies and partial subscapularis tears affect and alter the SSP muscle belly. We suggest early intervention for supraspinatus tears to avoid further fatty degeneration, as muscle atrophy and fatty degeneration progress in combination with the accompanying lesions.

## Introduction

Tears are more common in the supraspinatus (SSP) tendon than in any other tendons of the rotator cuff^1^. Chronic rotator cuff tears are associated with fatty degeneration of the muscles, as shown in animal models^2^, and fatty degeneration in the rotator cuff muscles has been related to the poor results after its repairs^3^. Regardless of sufficient tendon healing after tendon repair, high re-tear rates and long-term functional deficits of the muscle-tendon unit may persist from fatty degeneration or discordant neighbouring tendons^4^. Factors, which cause fatty degeneration, are still not clearly identified, but the multiple tendon ruptures could contribute to varying degrees of degeneration of the SSP tendon^5^. Defining factors related to the progress and timing of muscle degeneration are therefore potentially important for optimal surgical timing. Previous studies have shown that tear size and location are determining factors in the development of muscle degeneration^6^, but do not explain the mechanism of fatty degeneration.

The primary aim of this study was to evaluate the effects of infraspinatus and subscapularis tears, the neighbouring muscles of the supraspinatus, on the fatty degeneration of the SSP muscle. We hypothesised that accompanying lesions of subscapularis and infraspinatus muscles inversely affect the quality of the SSP muscle belly.

## Materials and Methods

After receiving Institutional Review Board approval and getting the informed consents of the patients, a retrospective review of patients admitted between August 2011 and December 2014 was performed. The primary patient inclusion criteria in this study were the presence of full-thickness SSP tendon tears and "substantial pain." The term "substantial pain" was coined by Mall *et al*^7^, defining any pain of ≥ 3 on a 10-point visual analogue pain scale that had lasted longer than six weeks, any pain considered to be greater than that normally experienced as part of daily living, any pain requiring the use of medications such as narcotics or non-steroidal anti-inflammatory drugs, or any pain that prompted a physician visit for evaluation.

During the examination, all patients were carefully questioned for the onset of new pain in the shoulder. We also identified and ruled out other potential reasons for shoulder pain such as arthritis or biceps tendon and acromioclavicular pathologies through radiographic and magnetic resonance imaging (MRI) techniques. Additional exclusion criteria were the presence of partial-thickness supraspinatus tears, any previous shoulder surgeries, and any subsequent trauma to the shoulder region after initiation of prior complaints. All the patients admitted on the stated dates were included in the study, provided they met the inclusion and exclusion criteria. The local hospital electronic database was used to review the patient records.

Fifty-eight patients with a full-thickness SSP tendon tear met the inclusion criteria, and they were evaluated for tear retraction, fatty degeneration, and other rotator cuff tendon pathologies. Accompanying lesions of the subscapularis and infraspinatus tendons were evaluated using MRI, which was performed using a 1.5T MRI unit [MAGNETOM Aera; Siemens Healthcare, Erlangen, Germany], and measurement of the images was performed in the provider’s image analysis software [syngo.via; Siemens Medical Solutions, Erlangen, Germany]. An experienced radiologist, with five years of subspecialty training in musculoskeletal radiology, and one orthopaedic surgeon, with six years of subspecialty training in sports medicine, especially shoulder surgery, analysed the results.

The size of the SSP tendon tear retraction was assessed using the method described by Meyer *et al*^8^. On the coronal sections, the length of the tear retraction. distance from the lateral edge of the humeral articular surface to the tendon end, was measured through the centre of the SSP tendon^8^. Also, correlations were performed between the exact tear retraction and SSP muscle area. The strength of the relationship was classified as follows: strong (r > 0.5), medium (0.3 < r < 0.5), small (0.1 < r < 0.3), or none (r < 0.1)^9^. Inter- and intraobserver reliability of the measurements was also calculated^10^. Furthermore, using the Goutallier classification as modified by Fuchs *et al*, the degree of fatty degeneration of SSP muscle was assessed^11, 12^. Stages were identified as follows: stage 0, normal muscle; stage 1, some fatty streaks; stage 2, manifest fatty degeneration but less fat than muscle; stage 3, as much fat as muscle; and stage 4, more fat than muscle. Muscle atrophy closely associated with fatty degeneration was also assessed by measuring the area of the SSP muscle as described by Thomazeau *et al*^13^. Thomazeau developed a reproducible and reliable method to define supraspinatus muscle atrophy with the help of MRI. They proposed a classification of supraspinatus belly atrophy based on the occupation ratio of the supraspinatus fossa.

Moreover, to be able to evaluate the atrophy of the supraspinatus muscle, occupation ratio calculation of the supraspinatus fossa needs to be performed. In the calculated ratio between 1.00 and 0.60, stage 1, it was determined that the muscle could be considered as normal or slightly atrophied. Meanwhile, in ratios between 0.60 and 0.40, this suggested moderate atrophy of stage 2; while values below 0.40 showed serious or severe atrophy of stage 313. Thus, we used quantitative values for statistical analysis, as it was impractical to perform correlations with the Goutallier scale. For clarification, the correlation between muscle atrophy and the degree of fatty degeneration was also investigated.

Infraspinatus and subscapularis tendon pathologies were also assessed, classifying tendons into the following scales as per Shi *et al*: normal cuff (1); tendinopathy (2); partial-thickness tendon tear (3); and full-thickness tendon tear (4)^14^. Thus, we analysed the effects of these two neighbouring tendon pathologies on the fatty degeneration of SSP tendon and assessed their relationship (Table I).

**Table I:** The effect of accompanying infraspinatus and subscapularis tendon pathologies on the supraspinatus (SSP) muscle belly

Tendon pathologies	N	SSP muscle belly	
**Infraspinatus**		**r**	**P**
Tendinosis	12	-0.565	< .05
Partial tear	13	-0.588	< .05
Full thickness tear	11	-0.603	< .05
**Subscapularis**		**r**	**P**
Tendinosis	17	-0.312	0.222
Partial thickness tear	13	-0.675	< .05
Full thickness tear	4	-0.738	0.325

r = correlation coefficient.

Statistical analyses were performed with SPSS software [version 17.0; SPSS Inc, Chicago, IL, USA]. One-way analysis of variance tests was used to determine which factors exerted a significant influence on SSP muscle tear from infraspinatus and subscapularis tendon pathologies.

## Results

Fifty-eight patients (34 women, 24 men) were included in this study. The average age of the patients was 62 years old (range, 41-68 years). The study showed that the average size of the full-thickness SSP tendon tear retraction was 19.0mm (range, 3mm and 41mm), and the average SSP muscle area was 247.6mm2 (range, 29–529).

The participating patients showed various levels of fatty degeneration according to the Goutallier classification: six patients scored a 0 (10%); 22 scored grade 1 (37.9%); 14 scored grade 2 (24.1%); nine scored grade 3 (15.5%); and seven scored grade 4 (12%). The Goutallier grades were also linked with the area of the SSP muscle belly (Table II). Therefore, it could be deduced that when the grade of fatty degeneration increased, the area of the SSP muscle belly decreased significantly (P < 0.05), demonstrating a strong negative correlation (correlation coefficient, r=0.623). Our results quantitatively demonstrated this relationship. It further showed that when the SSP tear size enlarged, the SSP muscle area decreased, but stayed at a steady state after a tear size of 25mm upwards (Fig. I).

**Fig. 1:**
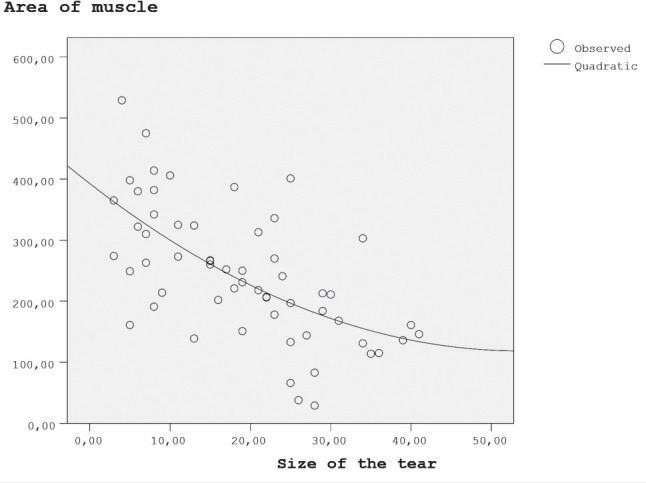
Parabolic curve becomes more horizontal to the x-axis, demonstaring a steady state.

**Table II:** Relation between the Goutallier classification and area of supraspinatus (SSP) muscle

Goutallier Classification		Area of SSP muscle		Levene test		Test of means	
	N	Mean	SD	F	Sig.	F	P
0	6	392.83	73.575				
1	22	295.04	74.919				
2	14	223.71	81.441	1.704	0.163	21.716	< .05*
3	9	175.00	29.681				
4	7	87.14	45.830				

SD = standard deviation; F = F-test of equality of variances; Sig. = significance

The effects of the accompanying infraspinatus and subscapularis tendon pathologies on the supraspinatus muscle belly were shown in Table I. For infraspinatus tendon pathologies, any degree of pathology was significantly associated with the SSP muscle area (P < 0.05) (Table I). The correlation coefficient was gradually increasing for all infraspinatus tendon pathologies, ranging from tendinosis to full-thickness tears. However, when we evaluated the subscapularis tendon, the correlation coefficient was strong for all tendon pathologies except for tendinosis. Despite the correlation between tendinosis and full-thickness tears, the statistical significance could not be achieved. Increasing the number of patients might be needed to verify the correlation to obtain statistical significance.

The interobserver and intraobserver reliabilities of the measurements were graded as excellent (intraclass correlation coefficients of 0.820 and 0.855, respectively).

## Discussion

Our results indicated that the accompanying pathologies of the infraspinatus and subscapularis tendons did affect the SSP muscle belly to different degrees. All infraspinatus tendon pathologies, including tendinosis and partial- and full-thickness tears, had strong gradually increasing correlation with the SSP fatty degeneration. In other words, SSP muscle atrophy gradually worsened when infraspinatus tendon pathology progressed from tendinopathy to full-thickness tears. Furthermore, it signified that all kinds of infraspinatus tendon pathologies affected the SSP muscle belly with gradually increasing correlation.

However, given the number of patients available for analysis, only partial-thickness tears of the subscapularis tendon demonstrated a significantly strong correlation. Tendinopathy and full-thickness tears of the subscapularis tendon did not have significant effects on the structure of SSP muscle, with a strong correlation as that between the infraspinatus and SSP tendons.

Previous studies also reported that fatty degeneration correlated with the pathology of the relevant rotator cuff tendon and that any disruption of the anterior SSP tendon was associated with more advanced SSP muscle degeneration^14, 15^. However, there was little information available regarding the effect of the adjacent rotator cuff tendons on the progression of the most often torn rotator cuff tendon, the SSP. Through our results, we could say that the whole rotator cuff bundle showed a particular synchron. It is well-known that the rotator cuff unit is a combination of four tendons, and the fibres of the SSP and infraspinatus tendons form a richly connected "footprint." It was recently suggested that the greater tuberosity was occupied by a substantial portion of the infraspinatus muscle^16^. Therefore, impairment of any of the neighbouring muscles could potentially affect the others inversely. In order to determine which muscles might affect the quality of the SSP, we investigated the impact of the tendons of the infraspinatus and subscapularis that were most proximate to the SSP, using MRI. For patients with underlying infraspinatus tendon pathologies, our study found that the highest correlation was seen for full-thickness tears of the infraspinatus tendon, where the patients would develop muscle atrophy and fatty degeneration to a greater degree. As a clinical relevance, the integrity of the infraspinatus and subscapularis tendons might be an important factor to consider for severe fatty degeneration and atrophy of the SSP muscle, in the outcome of surgical intervention.

Two distinct abnormalities had been identified in the literature: muscle atrophy with a reduced cross-sectional area of muscle or reduced muscle bulk; and fatty degeneration with the replacement of muscle fibres by fat^17^. Although highly correlated, no current evidence has shown exactly how these two entities are linked^11, 18^. In this study, we found a significantly close relationship between the muscle area and the Goutallier scale that precluded the simultaneous use of these two variables in the correlation analysis. Since a strong negative relationship existed between the SSP muscle area and the Goutallier scale, the SSP muscle area was used for statistical analysis (Table II).

We also analysed the correlation of the retraction of the SSP tendon tear and the degree of SSP muscle atrophy (Table III). Although predictable, our results quantitatively demonstrated this relationship, finding a strong correlation. However, interestingly, we noticed that when the SSP tear size enlarged, the SSP muscle area decreased but remained at a steady state after a tear size of 25mm upwards, demonstrating a plateau. This finding, which showed that the SSP muscle belly demonstrated a residual baseline state after the tear retraction reached 25mm, highlighted the importance of early intervention. As increasingly reported in the literature, early intervention for small- to medium-sized tears might protect the SSP muscle from fatty degeneration^19^. However, as a limitation, our study had a limited number of patients with tear sizes of more than 25mm.

**Table III:** Relation between the Goutallier classification and retraction of the tear

Factor Goutallier		Retraction of tear		Levene test		Test of means	
	N	Mean	SD	F	P-value	F	P
0	6	7,00	2.966				
1	22	14,00	8.423				
2	14	20,92	8.498	1,427	0.238	10,869	< .05*
3	9	26,88	9.816				
4	7	29,85	6.202				

SD = standard deviation; F = F-test of equality of variances

This study had other potential limitations, as well. Our patient group did not include any patients with a tear retraction greater than 41mm in length. Patients with larger tears would potentially represent massive retracted tears, and the inclusion of this group would affect the results. We also found another limitation in our conclusion that full-thickness tears of the subscapularis tendon did not affect the structure of SSP muscle significantly, as the sample size of the subscapularis full-thickness tears was small. Despite the correlation between tendinosis and full-thickness tears, the statistical significance could not be achieved. Increasing the number of patients in future studies would allow for better definite results.

## Conclusion

More attention should be paid to the accompanying pathologies of the infraspinatus tendon, especially for full-thickness tears, due to the increasing correlation with the supraspinatus muscle atrophy. Our study indicated that infraspinatus tendon pathologies, from tendinosis to full-thickness tears, affect and alter the SSP muscle belly gradually. Early intervention should be made for SSP tears to avoid further fatty degeneration, as muscle atrophy and fatty degeneration progress in combination with the accompanying lesions.
